# Not all *SCN1A* epileptic encephalopathies are Dravet syndrome: Early profound Thr226Met phenotype

**DOI:** 10.1212/WNL.0000000000004331

**Published:** 2017-09-05

**Authors:** Lynette G. Sadleir, Emily I. Mountier, Deepak Gill, Suzanne Davis, Charuta Joshi, Catherine DeVile, Manju A. Kurian, For the DDD Study, Simone Mandelstam, Elaine Wirrell, Katherine C. Nickels, Hema R. Murali, Gemma Carvill, Candace T. Myers, Heather C. Mefford, Ingrid E. Scheffer

**Affiliations:** From the Department of Paediatrics and Child Health (L.G.S., E.I.M.), University of Otago, Wellington, New Zealand; Department of Neurology (D.G.), University of Sydney, Australia; Department of Neurology (S.D.), Starship Children's Health, Auckland, New Zealand; Department of Neurology (C.J.), Children's Hospital Colorado, Anschutz Medical Campus, University of Colorado, Denver; Department of Neurology (C.D.V., M.A.K.), Great Ormond Street Hospital for Children; Developmental Neurosciences (M.A.K.), UCL Great Ormond Street Institute of Child Health, London; Wellcome Trust Sanger Institute (DDD Study Group), Hinxton, Cambridge, UK; Departments of Paediatrics and Radiology (S.M.), University of Melbourne; The Florey Institute of Neuroscience and Mental Health (S.M., I.E.S.); Department of Medical Imaging (S.M.), Royal Children's Hospital, Melbourne, Australia; Department of Neurology (E.W., K.C.N.), Mayo Clinic, Rochester, MN; Department of Neurology (H.R.M.), Marshfield Clinic, WI; Division of Genetic Medicine (G.C., C.T.M., H.C.M.), Department of Pediatrics, University of Washington, Seattle; and Departments of Medicine and Paediatrics (I.E.S.), University of Melbourne, Austin Health and Royal Children's Hospital, Australia.

## Abstract

**Objective:**

To define a distinct *SCN1A* developmental and epileptic encephalopathy with early onset, profound impairment, and movement disorder.

**Methods:**

A case series of 9 children were identified with a profound developmental and epileptic encephalopathy and *SCN1A* mutation.

**Results:**

We identified 9 children 3 to 12 years of age; 7 were male. Seizure onset was at 6 to 12 weeks with hemiclonic seizures, bilateral tonic-clonic seizures, or spasms. All children had profound developmental impairment and were nonverbal and nonambulatory, and 7 of 9 required a gastrostomy. A hyperkinetic movement disorder occurred in all and was characterized by dystonia and choreoathetosis with prominent oral dyskinesia and onset from 2 to 20 months of age. Eight had a recurrent missense *SCN1A* mutation, p.Thr226Met. The remaining child had the missense mutation p.Pro1345Ser. The mutation arose de novo in 8 of 9; for the remaining case, the mother was negative and the father was unavailable.

**Conclusions:**

Here, we present a phenotype-genotype correlation for *SCN1A*. We describe a distinct *SCN1A* phenotype, early infantile *SCN1A* encephalopathy, which is readily distinguishable from the well-recognized entities of Dravet syndrome and genetic epilepsy with febrile seizures plus. This disorder has an earlier age at onset, profound developmental impairment, and a distinctive hyperkinetic movement disorder, setting it apart from Dravet syndrome. Remarkably, 8 of 9 children had the recurrent missense mutation p.Thr226Met.

The finding of de novo *SCN1A* mutations in Dravet syndrome was paradigm shifting in our understanding of the etiology of the developmental and epileptic encephalopathies.^1,2^ Less commonly, inherited *SCN1A* mutations occur in the self-limiting familial epilepsy syndrome of genetic epilepsy with febrile seizures plus (GEFS+).^3^ Missense and truncation mutations are found in approximately equal frequency in Dravet syndrome, while GEFS+ is largely associated with missense mutations.^4^ Despite >1,200 reported *SCN1A* mutations and most patients having novel mutations, there are no clear phenotype-genotype correlations.

We have identified a distinctive *SCN1A* developmental and epileptic encephalopathy that is far more severe than Dravet syndrome and is associated with a recurrent missense mutation. It is characterized by early infantile seizure onset, profound intellectual disability, and a severe hyperkinetic movement disorder. This recurrent *SCN1A* mutation shows clear genotype-phenotype correlation.

## METHODS

Case 1 presented with an early infantile profound developmental and epileptic encephalopathy. Because he had hemiclonic seizures, the senior author (I.E.S.) requested *SCN1A* testing that revealed a de novo missense variant (Thr226Met). Because this phenotype was distinctive, we interrogated the Epilepsy Genetics Database at the University of Melbourne, which contained 981 individuals with solved and unsolved developmental and epileptic encephalopathies tested for *SCN1A* mutations. We searched for children with de novo *SCN1A* variants who presented at <4 months of age and had a movement disorder. All identified patients were included. This identified 3 additional cases: cases 2 and 6 with the Thr226Met variant and case 9 with the Pro1345Ser variant. Because 3 of the 4 cases had the Thr226Met variant, we searched our database and the literature for other cases with this variant and attempted to contact the authors for more phenotypic information. This resulted in an additional 2 cases (cases 3 and 4).^5^ The final 3 cases (cases 5, 7, and 8) were recruited when collaborators asked the first (L.G.S.) and senior (I.E.S.) authors about the role of the de novo *SCN1A* Thr226Met variant that they had identified in their patients who presented with an atypical phenotype for Dravet syndrome.

Epilepsy and medical history, neurologic examination, and MRI and EEG data were obtained for each patient. *SCN1A* mutations were identified by clinical or research testing; segregation testing in parents was possible for 8 individuals.

### Standard protocol approvals, registrations, and patient consents.

The Austin Health Human Research Ethics Committee and the New Zealand Health and Disability Ethics Committees approved the study. Informed consent was obtained for each patient from the parents or legal guardian. In addition, specific written consent for authorization of video use was obtained for the 3 cases in the video.

## RESULTS

We identified 9 (7 boys) unrelated children 3 to 12 years of age (table). They presented with seizures at a mean age of 9 weeks (range 6–12 weeks) with predominantly hemiclonic seizures (n = 6) but also with bilateral tonic-clonic seizures (followed 1 week later by hemiclonic seizures in 1 patient) or epileptic spasms. All children developed tonic-clonic seizures by 18 months. Eight had episodes of convulsive status epilepticus with onset between 8 weeks and 2 years (table). Myoclonic seizures (7 of 9), tonic seizures (5 of 9), and spasms (5 of 9) were also seen. Epilepsy was refractory to multiple antiepileptic medications in all cases, although 1 child became seizure free at 8 years. Five children had seizures associated with fever or illness, and 2 children had seizures exacerbated by high environmental temperature. There were no reports of vaccination triggering seizures. Other seizure triggers included auditory stimuli (2), excitement (3), feeding (2), and bowel movements (1).

**Table:**
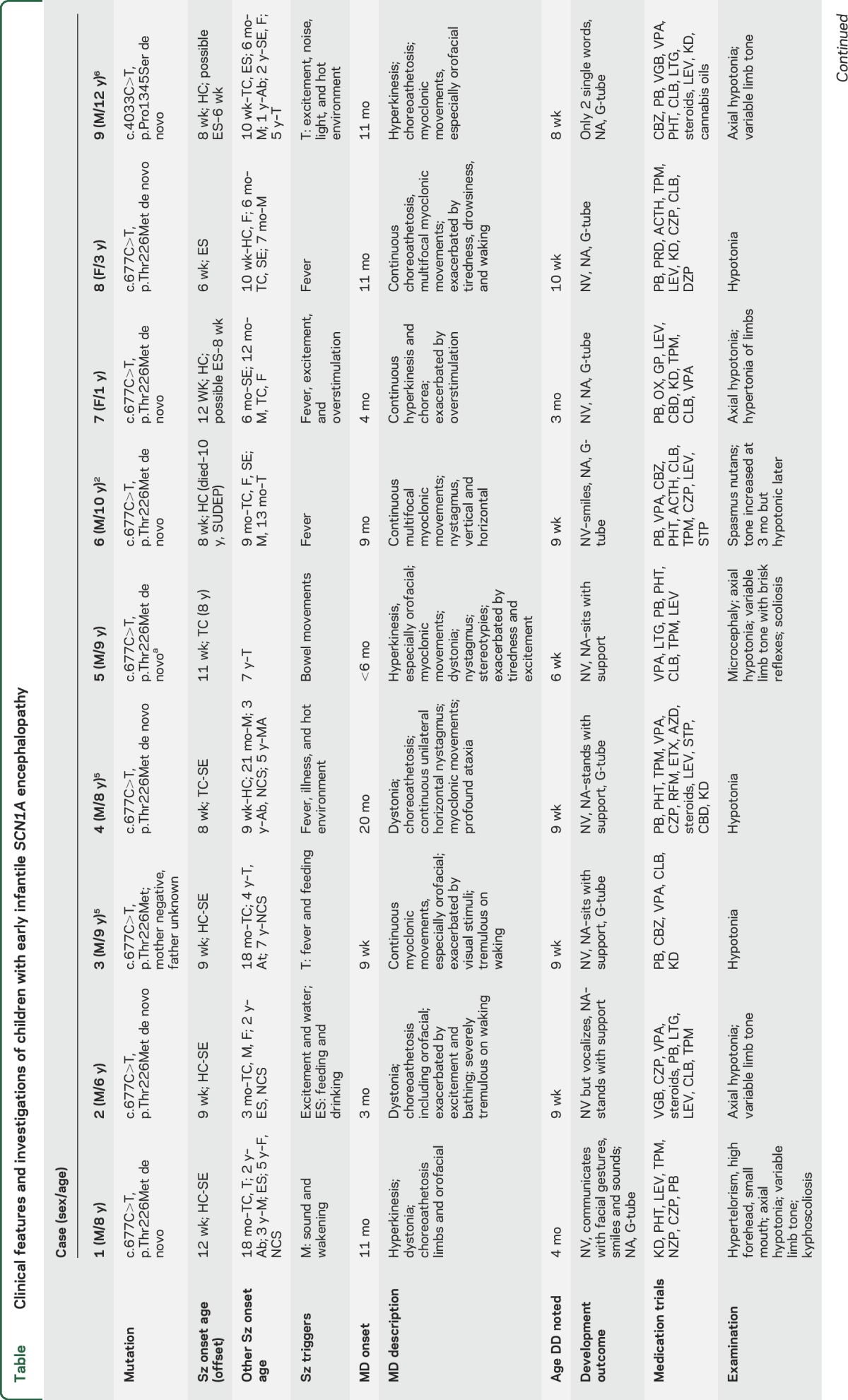

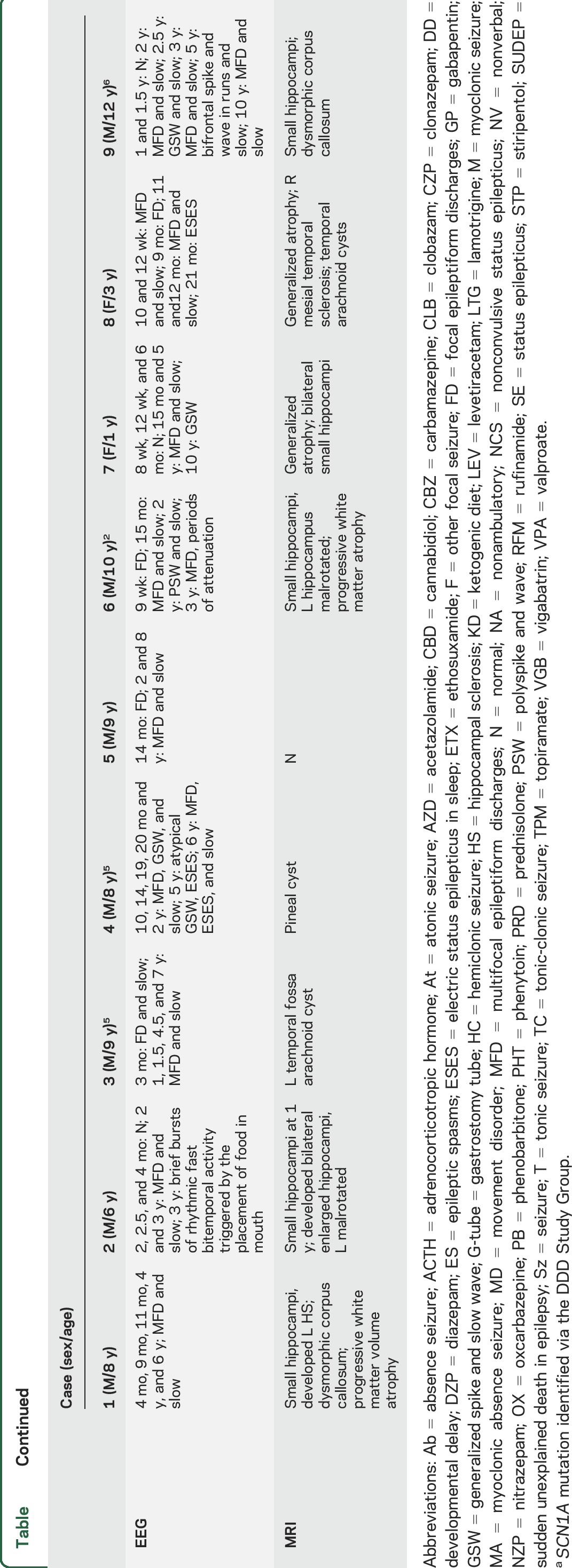
Clinical features and investigations of children with early infantile *SCN1A* encephalopathy

Initial development was considered normal by all parents, who gave reports of normal early developmental milestones. Delay was evident, however, by 6 to 16 weeks. At last review (range 3–12 years), 8 children were nonverbal, and 1 child spoke 2 single words. All were nonambulatory, and 7 required enteral feeding. The children had periods of regression or developmental plateauing resulting in profound intellectual disability. One child died at 10 years of sudden unexplained death in epilepsy.

All patients had a prominent movement disorder that developed within the first 2 years of life (9 weeks–20 months). Initially, they presented with subtle, low-amplitude myoclonic jerks. Over time, a hyperkinetic movement disorder comprising chorea, dystonia, and low-amplitude myoclonic movements was noted, particularly evident in the orofacial area (video at Neurology.org). Although not always present, the movement disorder was persistent and exacerbated on awakening and excitement. The abnormal movements were not seen during sleep. EEG recordings showed no epileptiform activity with these movements.

Eight of our 9 cases were trialed on antiepileptic drugs that could induce or exacerbate movement disorders or myoclonic seizures such as vigabatrin or sodium channel blockers (e.g., phenytoin, carbamazepine, oxcarbazepine, lamotrigine). In several cases, these drugs were begun well after the movement disorder had been recognized. Subsequent discontinuation of the medication had no effect on the movement disorder. There was also no clear exacerbation of their seizures with these medications; however, their seizures were so frequent that it may have been difficult to assess.

Neuroimaging was normal or showed nonspecific abnormalities. Four children had developmentally abnormal hippocampi; 2 had dysmorphic corpus callosal; and 2 had progressive white matter loss (table and figure). EEGs were initially normal in 3 children, but by 2 years of age, all had developed multifocal discharges and diffuse background slowing. Four children had generalized spike and slow wave or polyspike and slow wave.

**Figure:**
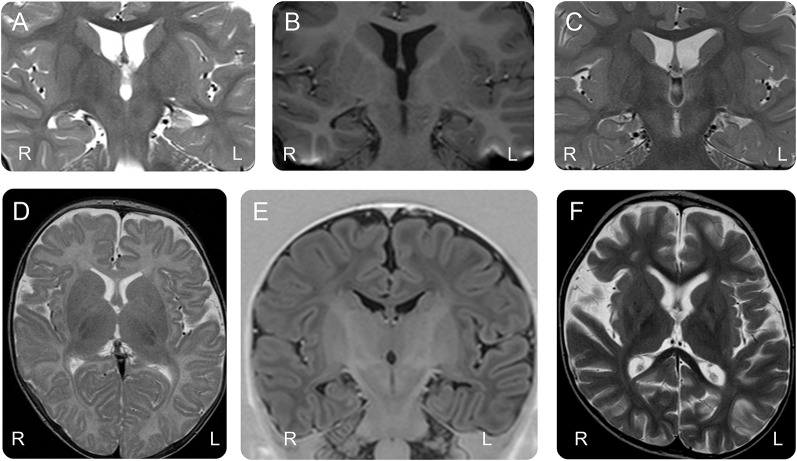
MRI in children with early infantile *SCN1A* encephalopathy (A–C) Coronal views in case 1 (3 years of age), case 9 (5 years of age), and case 2 (1 year of age) show bilateral small hippocampi. (A) Case 1 developed left hippocampal sclerosis. (C) Case 2 has a malrotated left hippocampus. (D, F) Axial T2 and coronal T1 images of case 9 show (D) normal white matter volume at 2 months of age with (F) moderately severe volume loss at 2 years. (E) Coronal views of the hippocampi at 2 months show bilateral small hippocampi with left hippocampal malrotation.

All patients had *SCN1A* mutations; 8 were confirmed de novo. The mother of case 3 was negative, and the father was unavailable. Cases 1, 2, and 9 were identified on molecular inversion probe–targeted resequencing^6^; cases 3, 4, and 8 were identified on clinical panels; case 5 was identified on whole-exome sequencing; and cases 6 and 7 were identified by *SCN1A* sequencing. No additional likely pathogenic variants were found in other genes in the cases that had targeted resequencing, clinical panels, or whole-exome sequencing. Eight children had the same missense mutation, p.Thr226Met, resulting from c.677C>T in exon 5. In silico tools suggested that it was deleterious (Grantham D = 81, Polyphen2 = 1, SIFT = 0, GERP = 5.77, CADD = 20.5). The ninth child had a de novo missense mutation, p.Pro1345Ser (c.4033C>T), which was pathogenic on in silico testing (Grantham D = 74, Polyphen2 = 1, SIFT = 0, GERP = 5.42, CADD = 23.7).

## CASE HISTORIES

### Case 1.

Case 1 is an 8-year-old boy who presented at 12 weeks of age with afebrile hemiclonic status epilepticus lasting 30 minutes. Frequent pharmacoresistant hemiclonic seizures lasting 1 minute to 6 hours occurred daily. The lateralization of the clonic activity varied and occasionally migrated from 1 upper limb, to hemiclonic, to both lower limbs, to hemiclonic on the other side. Bilateral tonic-clonic and tonic seizures began at 18 months, absence seizures at 2 years, and myoclonic seizures or epileptic spasms at 3 years. By 5 years, he developed focal impaired awareness seizures that could progress to nonconvulsive status epilepticus lasting hours, frequent nocturnal tonic seizures, and weekly series of epileptic spasms. Seizures were triggered by waking but not by illness or fever. He has had multiple hospital admissions for status epilepticus and poor seizure control, the longest a 3-month admission at 18 months. Trials of phenobarbital, phenytoin, nitrazepam, clonazepam, levetiracetam, and topiramate were ineffective. The ketogenic diet was initiated at 4 years and resulted in improved control with cessation of myoclonic seizures. By 8 years, he was on ketogenic diet monotherapy and continued to have weekly tonic-clonic, focal impaired awareness, and tonic seizures in sleep and required admission every few months.

From birth, he had significant early feeding difficulties. Developmental milestones were otherwise normal with good head control, smiling, and fixing and following by 6 weeks. From 4 months, his development plateaued and fluctuated over the next few years with regression between 3 and 4 years. He lost eye contact, smiling, vocalization, head control, and the ability to roll over. He required a gastrostomy for feeding. When he was initiated on the ketogenic diet at 4.5 years, he had a marked improvement in alertness and developmental gains. At 8 years, he smiled, laughed, and vocalized vowel sounds but was nonverbal. He did not feed orally. He was able to roll over but could not sit.

He developed a hyperkinetic movement disorder by his first birthday consisting of choreiform movements including the orofacial area, dystonic posturing of the upper and lower limbs, and myoclonus without EEG correlate (video). Video monitoring at 16 months confirmed that all the abnormal movements did not have an epileptiform correlate. He has normal tone in his limbs with reduced truncal tone. He has hypertelorism, a high forehead, a small mouth, a normal head circumference, and a mild kyphoscoliosis. EEGs from 4 months showed multifocal epileptiform activity with background slowing. At 4 months, MRI brain revealed bilateral small hippocampi with a dysmorphic corpus callosum. By 3 years, he had developed left hippocampal sclerosis and progressive white matter atrophy (figure, A).

### Case 2.

Case 2 is a 6-year-old boy who presented at 9 weeks with 5 independent afebrile right and left hemiclonic seizures lasting from 1 to 20 minutes over a 2-hour period that were treated with buccal and intravenous midazolam and intravenous phenytoin. Occasional hemiclonic seizures continued despite the introduction of phenobarbital and topiramate. He developed myoclonic, focal impaired awareness and bilateral tonic-clonic seizures by 13 weeks. The seizures were not associated with fever but were more likely to occur on waking. At 13 weeks, he developed a periodic movement disorder initially lasting 10 to 30 minutes consisting of choreoathetosis, dystonia, and small-amplitude myoclonic jerks, which his parents called “dancing.” This was triggered by waking and excitement, particularly bathing. His movement disorder progressed over the next 18 months so that by 2 years it occurred almost continuously, although it settled in sleep. He did, however, have massive myoclonic jerks without EEG correlate on awakening. His epilepsy was refractory to sodium valproate, lamotrigine, and levetiracetam. From 2 years, he had infrequent episodes of focal nonconvulsive status epilepticus, characterized by loss of awareness, eye deviation, and facial twitching, lasting up to 2 hours. The EEG showed high-voltage rhythmic focal delta in the centro-parietal region. At 2.5 years, he developed flexor epileptic spasms when eating or drinking. The spasms occurred every 10 to 20 seconds for 3 minutes shortly after food or liquid was put in his mouth and interfered with his ability to chew and swallow. The spasms were controlled for 2 months but then relapsed and are ongoing. By 4 years, the massive myoclonic jerks were awakening him overnight, which triggered his movement disorder and prevented him from sleeping, posing a major problem for the family’s quality of life.

His development was normal at 9 weeks; he smiled and fixed and followed by 6 weeks. Within a week of seizure onset, he regressed with loss of visual fixation and head control. By 5 months, he was rolling over, and by 8 months, he was sitting and finger-feeding. After 9 months, his development plateaued with a period of regression at 2 years. At 6 years, he vocalizes but is nonverbal, nor can he sit or walk independently.

On examination, he was not dysmorphic and had almost continuous choreiform movements involving the limbs and orofacial region (video). His limb tone was variable with axial hypotonia. Early EEGs between 10 weeks and 4 months were normal, but multifocal epileptiform abnormalities had developed by 2 years. MRI at 1 year showed bilateral small hippocampi with left hippocampal malrotation (figure, C).

## DISCUSSION

*SCN1A* is the most relevant epilepsy gene. Despite considerable effort, no phenotype-genotype correlation has been shown for the hundreds of *SCN1A* mutations identified, with the majority associated with Dravet syndrome and a small proportion with GEFS+.^4^ Here, we show a phenotype-genotype correlation for *SCN1A* describing a distinct *SCN1A* phenotype, early infantile *SCN1A* encephalopathy, that is far more severe than Dravet syndrome. We bring together novel cases and reanalyze the phenotype of reported cases with the recurrent mutation to identify a distinctive entity of early-onset developmental and epileptic encephalopathy with profound impairment and a prominent movement disorder.

Early infantile *SCN1A* encephalopathy can be readily distinguished from Dravet syndrome by several features. It has a younger age at onset, beginning at <3 months compared with the typical seizure onset age range of 4 to 15 months in Dravet syndrome. It is associated with profound developmental impairment rather than the severe to mild intellectual disability usually seen in Dravet syndrome. Infantile movement disorders are not part of the Dravet phenotype, whereas our patients have a distinctive movement disorder with choreoathetosis, dystonia, and perioral hyperkinesia. Other features differentiating early infantile *SCN1A* encephalopathy from Dravet syndrome are epileptic spasms, which are not seen in Dravet syndrome. Tonic seizures are described in adults with Dravet syndrome but are not part of the childhood phenotype.^7^

We describe that the Thr226Met mutation is associated with a distinctive profound *SCN1A* encephalopathy. Three of the 8 patients with Thr226Met (cases 3, 4, and 6) have been previously reported as having Dravet syndrome.^2,5^ Case 6 was identified in 2007 in our large study delineating the phenotypic spectrum of Dravet syndrome.^2^ Cases 3 and 4 were drawn from a 2014 study of sleep problems associated with *SCN1A*-confirmed Dravet syndrome,^5^ but the phenotype was not described in the report. The phenotype, early infantile *SCN1A* encephalopathy, was not yet recognized, and Dravet-like features such as hemiclonic seizures led to the diagnosis of Dravet syndrome. We have reanalyzed the phenotype of these patients, and all share a homogeneous profound phenotype.

In addition, there are 2 further individuals reported with the Thr226Met mutation.^2,8^ One is a child described as having progressive myoclonus epilepsy; however, the limited available clinical information suggests that the phenotype of this child could be consistent with early infantile *SCN1A* encephalopathy.^8^ Overlap between the movement disorder and a progressive myoclonus epilepsy could be challenging to distinguish, especially given that the patient was middle-aged at the time of review. The remaining child presented with febrile status epilepticus at 6 months.^2^ However, she then developed extensor epileptic spasms at 10 months, which would be extraordinary for Dravet syndrome.^7^ This patient was not reported to have a movement disorder, but at 9 months, she had continuous myoclonic activity diagnosed as nonconvulsive status epilepticus. This could be a differential diagnosis of the movement disorder in our patients, but overall, she had better developmental progress. She also had an *SCN9A* variant (p.W1538R) that may have modified her phenotype.^9^ Perhaps this caused loss of function of *SCN9A*, which in some way modified the functional alteration due to the *SCN1A* Thr226Met mutation.

The Thr226Met mutation is not the only *SCN1A* mutation described at this specific codon. There are 2 reports of missense *SCN1A* mutations at codon c.677 that result in a different amino acid change to Thr226Arg or Thr226Lys.^10,11^ Not enough clinical information is provided in the reports to allow assessment of whether these children have a similarly profound phenotype.

A Japanese group has previously described a single case of infantile epileptic encephalopathy with a hyperkinetic movement disorder and hand stereotypies associated with a different novel *SCN1A* mutation.^12,13^ This Japanese child's presentation sounds like early infantile *SCN1A* encephalopathy in terms of seizure onset age of 8 weeks; seizure types included epileptic spasms, hemiclonic seizures, myoclonic seizures, generalized tonic-clonic seizures, and status epilepticus, with profound impairment. The child also had an early-onset severe hyperkinetic movement disorder and a different *SCN1A* missense mutation (c.1264 G>T, p.Val422Leu) from that found in our cohort.^12,13^

Despite there being >1,200 different reported *SCN1A* mutations, only 18% of mutations are recurrent.^4^ Mutations associated with GEFS+ are more likely to have a lower Grantham score than those that cause Dravet phenotypes.^4,14^ Missense mutations in the pore region lead to complete loss of function, similar to haploinsufficiency, but are seen in both Dravet syndrome (54%) and GEFS+ (31%).^4^ It is therefore not usually possible to predict an individual's phenotype on the basis of their specific *SCN1A* mutation. Here, however, we have identified a severe-striking *SCN1A* phenotype, early infantile *SCN1A* encephalopathy, that has a recurrent mutation at c.677 in almost all cases. Somewhat counterintuitively, this more severe phenotype is associated with missense, rather than truncation, mutations.

There is increasing recognition of the overlap of developmental epileptic encephalopathies and movement disorders. Severe hyperkinetic (dystonia, choreoathetosis, myoclonus) movements have been described in a range of genetic encephalopathies caused by *SCN2A*, *SCN8A*, *FOXG1*, *STXBP1*, *GNAO1*, *ARX*, and *DNM1*, among others.^12,15–17^ Distinguishing a movement disorder from seizures may affect therapeutic approaches in terms of when to introduce antiepileptic therapy or other treatment modalities.

There is a large group of early-onset developmental and epileptic encephalopathies defined by their onset age of <3 months, often not fitting within a recognized epilepsy syndrome. These disorders have been recently associated with a large, heterogeneous range of genetic etiologies.^18^ They share many common features such as multiple seizure types, profound to severe developmental delay, and movement disorders. It is unlikely that the seizures or the treatment per se cause the profound developmental impairment or movement disorders, given that a similar spectrum of seizure types with onset in later infancy or childhood does not have the same developmental sequelae. For example, infants with the self-limited “benign” early infantile seizure disorders may have frequent seizures with an abnormal EEG but without the long-term sequelae. Thus, we hypothesize that specific *SCN1A* mutations cause the overall phenotype. However, it is conceivable that this complex phenotype could be due to frequent seizures at a younger age.

*SCN2A* and *SCN8A* encephalopathies both are associated with gain-of-function mutations and respond well to sodium channel blockers.^15,16^ Here, we show that *SCN1A* also contributes to the severe early infantile–onset developmental encephalopathies, but we do not yet understand why this recurrent mutation causes this profound phenotype. It is interesting to speculate that it possibly is associated with a gain of function akin to *SCN2A* and *SCN8A*, but functional studies are required to prove the mechanism of this devastating disorder.

## Supplementary Material

Video

Coinvestigators

## GLOSSARY

GEFS+: genetic epilepsy with febrile seizures plus
