# Supplementary material for: Not all SCN1A epileptic encephalopathies are Dravet syndrome: Early profound Thr226Met phenotype
Source: Neurology. 2017 Sep 5;89(10):1035–42. doi: 10.1212/WNL.0000000000004331 (PMC5589790; doi:10.1212/WNL.0000000000004331)
Supplement: Video [file supp_89_10_1035__index.html]

Not all SCN1A epileptic encephalopathies are Dravet syndrome — Video 

# Not all *SCN1A* epileptic encephalopathies are Dravet syndrome

## Video

**Neurology® data supplements are not copyedited before publication. Published editorials and translations have been copyedited.  
 © 2017 American Academy of Neurology.  
  
 Files in this Data Supplement:**

- Video Legend - Microsoft Word file
- Video - .mp4 file
