# Supplementary material for: Not all SCN1A epileptic encephalopathies are Dravet syndrome: Early profound Thr226Met phenotype
Source: Neurology. 2017 Sep 5;89(10):1035–42. doi: 10.1212/WNL.0000000000004331 (PMC5589790; doi:10.1212/WNL.0000000000004331)
Supplement: Video [file supp_WNL.0000000000004331_Video_Legend.docx]

**Video Legend:** This video demonstrates the hyperkinetic movement disorder in three of the cases. Left is case 1, middle is case 2 and right is case 9.
