# Supplementary material for: Not all SCN1A epileptic encephalopathies are Dravet syndrome: Early profound Thr226Met phenotype
Source: Neurology. 2017 Sep 5;89(10):1035–42. doi: 10.1212/WNL.0000000000004331 (PMC5589790; doi:10.1212/WNL.0000000000004331)
Supplement: Coinvestigators [file supp_WNL.0000000000004331_Coinvestigators.pdf]

COINVESTIGATORS

A. Paul Bevan (Wellcome Trust Sanger Institute, DECIPHER Team); Abhijit Dixit (Nottingham University Hospitals NHS Trust, Recruiting Consultant Clinical Geneticist); Abigail Pridham (Oxford Radcliffe Hospitals NHS Trust, Research Nurse/Gene tic Counsellors); Adrian R. Tivey (Wellcome Trust Sanger Institute & University of Oxford, DDD Informatics Team); Ajoy Sarkar (Nottingham University Hospitals NHS Trust, Recruiting Consultant Clinical Geneticist); Alan Donaldson (Bristol Genetics Service, Patient recruitment and phenotyping); Alan Fryer (Royal Liverpool Children's Hospital, Recruiting Consultant Clinical Geneticist); Alejandro Sifrim (Wellcome Trust Sanger Institute & University of Oxford, DDD Analysis Team); Alex Henderson (Newcastle upon Tyne Hospitals NHS Foundation Trust & International Centre for Life, Recruiting Consultant Clinical Geneticist); Alex Magee (Belfast City Hospital, Recruiting Consultant Clinical Geneticist); Alexis Duncan (Yorkhill Hospital, Research Nurse/Genetic Counsellors); Alison Kraus (Chapel Allerton Hospital, Recruiting Consultant Clinical Geneticist); Alison Male (Great Ormond St Hospital, Patient recruitment and phenotyping); Alison Ross (North of Scotland Regional Genetics Service, Recruiting Consultant Clinical Geneticist); Amanda Collins, (Princess Anne Hospital, Salisbury District Hospital, Recruiting Consultant Clinical Geneticists); Anand Saggar (University of London, Recruiting Consultant Clinical Geneticist); Andrea Coates (Chapel Allerton Hospital, Diagnostic Laboratory Scientist); Andrea Nemeth (Oxford Regional Genetics Service, Patient recruitment and phenotyping); Andrew Fry (University Hospital of Wales & Glan Clywd Hospital, Patient recruitment and phenotyping); Andrew Green (Our Lady’s Children’s Hospital, Patient recruitment and phenotyping); Andrew Jackson (University of Edinburgh, Western General Hospital, Patient recruitment and phenotyping); Andrew Norman (Birmingham Women's Hospital, Recruiting Consultant Clinical Geneticist); Angela Barnicoat (Great Ormond Street Hospital, Patient recruitment and phenotyping); Angela Brady (Northwick Park Hospital & St Mark's Hospital, Recruiting Consultant Clinical Geneticist); Angela Douglas, (Royal Liverpool Children's Hospital, Diagnostic Laboratory Scientist); Angus Clarke (University Hospital of Wales & Glan Clywd Hospital, Patient recruitment and phenotyping); Angus Dobbie (Chapel Allerton Hospital, Patient recruitment and phenotyping); Ann Selby (Nottingham University Hospitals NHS Trust, Research Nurse/Genetic Counsellor); Anna Middleton (Wellcome Trust Sanger Institute & University of Oxford, DDD Ethics, Social Science and Policy Team); Anne Lampe (University of Edinburgh, Western General Hospital, Recruiting Consultant Clinical Geneticists); Anneke Seller (Oxford Radcliffe Hospitals NHS Trust, Diagnostic Laboratory Scientist); Annie Procter (University Hospital Of Wales & Glan Clwyd Hospital, Recruiting Consultant Clinical Geneticist) Karenza Evans (University Hospital Of Wales & Glan Clwyd Hospital, Research Nurse/Genetic Counsellor); Anthony Vandersteen (Northwick Park Hospital & St Mark's Hospital, Recruiting Consultant Clinical Geneticist); Astrid Weber (Royal Liverpool Children's Hospital, Recruiting Consultant Clinical Geneticist); Audrey Smith (Chapel Allerton Hospital, Recruiting Consultant Clinical Geneticist); Audrey Torokwa (Princess Anne Hospital, Salisbury District Hospital, Research Nurse/Genetic Counsellor); Beckie Kaemba (Leicester Royal Infirmary, Research Nurse/Genetic Counsellor); Becky Treacy (Cambridge University Hospitals NHS Foundation Trust, Diagnostic Laboratory Scientist); Beiyuan Fu (Wellcome Trust Sanger Institute & University of Oxford, WTSI FISH Team); Ben Hutton (Wellcome Trust Sanger Institute, Sample and data processing); Birgitta Bernhard (Northwick Park Hospital & St Mark's Hospital, Recruiting Consultant Clinical Geneticist); Bronwyn Kerr (St Mary’s Hospital & Central Manchester University Hospitals NHS Foundation Trust, Recruiting Consultant Clinical Geneticist); Bruce Castle (Royal Devon & Exeter Hospital, Recruiting Consultant Clinical Geneticists); Carina Donnelly (St Mary’s Hospital & Central Manchester University Hospitals NHS Foundation Trust, Research Nurse/Genetic Counsellors); Carol Gardiner (Yorkhill Hospital, Recruiting Consultant Clinical Geneticist); Carol Scott (Wellcome Trust Sanger Institute, WTSI Pipelines Staff); Carole Brewer (Royal Devon & Exeter Hospital, Recruiting Consultant Clinical Geneticists); Caroline F. Wright (Wellcome Trust Sanger

Institute & University of Oxford, DDD Management Team); Caroline Langman (Guy's Hospital, Research Nurse/Genetic Counsellors); Caroline Ogilvie (Guy's Hospital, Diagnostic Laboratory Scientist); Caroline Pottinger (Glan Clwyd Hospital, Patient recruitment and phenotyping); Carolyn Tysoe (Royal Devon & Exeter Hospital, Diagnostic Laboratory Scientist); Cat Taylor (Sheffield Children's NHS Trust, Research Nurse/Genetic Counsellors); Catherine McWilliam (North of Scotland Regional Genetics Service, Patient recruitment and phenotyping); Charles Shaw-Smith (Royal Devon & Exeter Hospital, Recruiting Consultant Clinical Geneticists); Charu Deshpande (Guy's Hospital, Recruiting Consultant Clinical Geneticist); Cheryl Longman (West of Scotland Regional Genetics Service, Patient recruitment and phenotyping); Cheryl Sequeira (Northwick Park Hospital & St Mark's Hospital, Research Nurse/Genetic Counsellor); Chirag Patel (Birmingham Women's Hospital, Recruiting Consultant Clinical Geneticist); Chris Bennett (Chapel Allerton Hospital, Recruiting Consultant Clinical Geneticist); Chris Nellåker, (University of Oxford & John Radcliffe Hospital, Method development and data analysis); Christopher Wragg (St Michael's Hospital, Diagnostic Laboratory Scientist); Claire Kirk (Belfast City Hospital, Research Nurse/Genetic Counsellor); Claire Turner (Royal Devon & Exeter Hospital, Recruiting Consultant Clinical Geneticists); Daniel King (Wellcome Trust Sanger Institute & University of Oxford, DDD Analysis Team); Daniel M. Barrett (Wellcome Trust Sanger Institute & University of Oxford, DDD Laboratory Team); Daniel Perrett (Wellcome Trust Sanger Institute, Sample and data processing); Daniela T. Pilz (University Hospital Of Wales & Glan Clwyd Hospital, Recruiting Consultant Clinical Geneticist); Danielle Walker (Wellcome Trust Sanger Institute, WTSI Pipelines Staff); David Baty, (Ninewells Hospital, Diagnostic Laboratory Scientist); David Bohanna (Birmingham Women's Hospital, Diagnostic Laboratory Scientist); David Bourn, (Newcastle upon Tyne Hospitals NHS Foundation Trust & International Centre for Life, Diagnostic Laboratory Scientist); David Goudie (Ninewells Hospital, Recruiting Consultant Clinical Geneticist); David J. Bunyan (Princess Anne Hospital, Salisbury District Hospital, Diagnostic Laboratory scientists); David Jones (Wellcome Trust Sanger Institute, WTSI Pipelines Staff); David Moore (University of Edinburgh, Western General Hospital, Diagnostic Laboratory Scientist); David R. FitzPatrick (University of Edinburgh, Western General Hospital, Recruiting Consultant Clinical Geneticists); David R. FitzPatrick (Wellcome Trust Sanger Institute & University of Oxford, DDD Management Team); Debbie Rice (Ninewells Hospital, Research Nurse/Genetic Counsellor); Debbie Shears (Oxford Radcliffe Hospitals NHS Trust, Recruiting Consultant Clinical Geneticist); Deirdre Cilliers (Oxford Regional Genetics Service, Patient recruitment and phenotyping); Deirdre Donnelly (Belfast City Hospital, Recruiting Consultant Clinical Geneticist); Denise Williams (Birmingham Women's Hospital, Recruiting Consultant Clinical Geneticist); Derek Lim (Birmingham Women's Hospital, Recruiting Consultant Clinical Geneticist); Dhavendra Kumar, Emma McCann (University Hospital Of Wales & Glan Clwyd Hospital, Recruiting Consultant Clinical Geneticist); Dian Donnai (St Mary’s Hospital, Patient recruitment and phenotyping); Diana Baralle (Princess Anne Hospital, Salisbury District Hospital, Recruiting Consultant Clinical Geneticists); Diana Johnson (Sheffield Children's NHS Trust, Recruiting Consultant Clinical Geneticist); Diana Rajan (Wellcome Trust Sanger Institute & University of Oxford, DDD Laboratory Team); Diana Wellesley (Princess Anne Hospital, Salisbury District Hospital, Recruiting Consultant Clinical Geneticists); Dominic J McMullan (Birmingham Women's Hospital , Diagnostic Laboratory Scientist); Douglas Simpkin (Wellcome Trust Sanger Institute, WTSI Pipelines Staff); Dragana Josifova (Guy's Hospital, Recruiting Consultant Clinical Geneticist); Dylan de Vries (Method development and data analysis) Eamonn Sheridan (Chapel Allerton Hospital, Patient recruitment and phenotyping); Eddy Maher, (University of Edinburgh, Western General Hospital, Diagnostic Laboratory Scientist); Edward Blair (Oxford Radcliffe Hospitals NHS Trust, Recruiting Consultant Clinical Geneticist); Eileen Roberts (St Michael's Hospital, Diagnostic Laboratory Scientist); Elena Chatzimichali (Wellcome Trust Sanger Institute, Sample and data processing); Elena Prigmore (Wellcome Trust Sanger Institute & University of Oxford, DDD Laboratory Team); Elisabeth Rosser (Great Ormond Street Hospital, Recruiting Consultant Clinical Geneticist); Elizabeth Jones (St Mary’s Hospital & Central Manchester University Hospitals NHS

Foundation Trust, Recruiting Consultant Clinical Geneticist); Elizabeth Sweeney (Royal Liverpool Children's Hospital, Recruiting Consultant Clinical Geneticist); Emily Wilkinson (Wellcome Trust Sanger Institute, Sample and data processing); Emma Gray (Wellcome Trust Sanger Institute, WTSI Pipelines Staff); Emma Hobson (Chapel Allerton Hospital, Recruiting Consultant Clinical Geneticist); Emma Kivuva (Royal Devon & Exeter Hospital, Recruiting Consultant Clinical Geneticists); Emma Miles (St Mary’s Hospital & Central Manchester University Hospitals NHS Foundation Trust, Diagnostic Laboratory scientists); Emma Shearing (Sheffield Children's NHS Trust, Diagnostic Laboratory Scientist); Emma Wakeling (Northwick Park Hospital & St Mark's Hospital, Recruiting Consultant Clinical Geneticist); Esther Kinning (Yorkhill Hospital, Recruiting Consultant Clinical Geneticist); Eugene Bragin (Wellcome Trust Sanger Institute, DECIPHER Team); Eve L. Coomber (Wellcome Trust Sanger Institute & University of Oxford, DDD Model Organisms); Fentang Yang (Wellcome Trust Sanger Institute & University of Oxford, WTSI FISH Team); Fiona Connell (Guy's Hospital, Recruiting Consultant Clinical Geneticist); Fiona Stewart (Belfast City Hospital, Recruiting Consultant Clinical Geneticist); Frances Elmslie (University of London, Recruiting Consultant Clinical Geneticist); Frances Flinter (Guy's Hospital, Recruiting Consultant Clinical Geneticist); G. Jawahar Swaminathan (Wellcome Trust Sanger Institute, DECIPHER Team); Gail Kirby (Birmingham Women's Hospital, Research Nurse/Genetic Counsellor); Gareth Cross (Nottingham University Hospitals NHS Trust, Diagnostic Laboratory Scientist); Gemma Devlin (Royal Devon & Exeter Hospital, Research Nurse/Genetic Counsellor); Geoff Woods (Cambridge University Hospitals NHS Foundation Trust, Recruiting Consultant Clinical Geneticist); Georgina Hollingsworth (Great Ormond Street Hospital, Research Nurse/Genetic Counsellors); Gillian Roberts (Royal Liverpool Children's Hospital, Research Nurse/Genetic Counsellor); Gordon Lowther (Yorkhill Hospital, Diagnostic Laboratory Scientist); Harinder Gill (Our Lady’s Children’s Hospital, Patient recruitment and phenotyping); Hayley Archer (University Hospital Of Wales & Glan Clwyd Hospital, Recruiting Consultant Clinical Geneticist); Helen Cox (Birmingham Women's Hospital, Recruiting Consultant Clinical Geneticist); Helen Firth (Cambridge University Hospitals NHS Foundation Trust, Recruiting Consultant Clinical Geneticist); Helen Kingston (St Mary’s Hospital, Patient recruitment and phenotyping); Helen Murphy (St Mary’s Hospital, Patient recruitment and phenotyping); Helen Stewart (Oxford Radcliffe Hospitals NHS Trust, Recruiting Consultant Clinical Geneticist); Helen V. Firth (Wellcome Trust Sanger Institute & University of Oxford, DDD Management Team); Hellen Purnell (Oxford Radcliffe Hospitals NHS Trust, Research Nurse/Genetic Counsellors); Hood Mugalaasi (University Hospital Of Wales & Glan Clwyd Hospital, Diagnostic Laboratory Scientist); Ian Ellis (Royal Liverpool Children's Hospital, Patient recruitment and phenotyping); Ingrid Scurr (Bristol Genetics Service, Patient recruitment and phenotyping); Ingrid Simonic (Cambridge University Hospitals NHS Foundation Trust, Diagnostic Laboratory Scientist); Irina Colgiu (Wellcome Trust Sanger Institute, Sample and data processing); Jacqueline Eason (Nottingham University Hospitals NHS Trust, Recruiting Consultant Clinical Geneticist); Jana Awada (Wellcome Trust Sanger Institute, Method development and data analysis); Jane Hurst (Great Ormond Street Hospital, Recruiting Consultant Clinical Geneticist); Jeffrey C. Barrett (Wellcome Trust Sanger Institute & University of Oxford, DDD Analysis Team); Jenny Morton (Birmingham Women's Hospital, Recruiting Consultant Clinical Geneticist); Jenny Thomson (Chapel Allerton Hospital, Recruiting Consultant Clinical Geneticist); Jeremy McRae (Wellcome Trust Sanger Institute & University of Oxford, DDD Analysis Team); Jill Clayton-Smith (St Mary’s Hospital & Central Manchester University Hospitals NHS Foundation Trust, Recruiting Consultant Clinical Geneticist); Joan Paterson (Cambridge University Hospitals NHS Foundation Trust, Recruiting Consultant Clinical Geneticist); Joanna Jarvis (Birmingham Women's Hospital, Recruiting Consultant Clinical Geneticist); Joanna Kaplanis (Wellcome Trust Sanger Institute, Method development and data analysis); Joanna Poulton (Oxford Regional Genetics Service, Patient recruitment and phenotyping); John Burn (Newcastle upon Tyne Hospitals NHS Foundation Trust & International Centre for Life, Recruiting Consultant Clinical Geneticist); John Burton (Wellcome Trust Sanger Institute, WTSI Pipelines Staff); John Dean (North of Scotland Regional Genetics Service, Recruiting Consultant

Clinical Geneticist); John Tolmie (Yorkhill Hospital, Recruiting Consultant Clinical Geneticist); Jonathan Berg (Ninewells Hospital, Recruiting Consultant Clinical Geneticist); Jonathan Roberts (Cambridge University Hospitals NHS Foundation Trust, Research Nurse/Genetic Counsellor); Jonathon Waters (Great Ormond Street Hospital, Diagnostic Laboratory Scientist); Josh Randall (Wellcome Trust Sanger Institute, Sample and data processing); Judith Goodship (Newcastle upon Tyne Hospitals NHS Foundation Trust & International Centre for Life, Recruiting Consultant Clinical Geneticist); Julia Rankin (Royal Devon & Exeter Hospital, Recruiting Consultant Clinical Geneticists); Julian Sampson (University Hospital of Wales & Glan Clwyd Hospital, Patient recruitment and phenotyping); Julie Phipps (Oxford Radcliffe Hospitals NHS Trust, Research Nurse/Genetic Counsellors); Julie Vogt (Birmingham Women's Hospital, Recruiting Consultant Clinical Geneticist); Kai-Ren Ong (Birmingham Women’s Hospital, Patient recruitment and phenotyping); Karen Marks, (University of London, Diagnostic Laboratory Scientist); Kate Brunstrom (Great Ormond Street Hospital, Research Nurse/Genetic Counsellors); Kate Chandler (St Mary’s Hospital & Central Manchester University Hospitals NHS Foundation Trust, Recruiting Consultant Clinical Geneticist); Kate Tatton-Brown (University of London, Recruiting Consultant Clinical Geneticist); Kath Smith (Sheffield Children's NHS Trust, Diagnostic Laboratory Scientist); Katherine I. Morley (Wellcome Trust Sanger Institute & University of Oxford, DDD Analysis Team); Katherine Lachlan, I. Karen Temple (Princess Anne Hospital, Salisbury District Hospital, Recruiting Consultant Clinical Geneticists); Katherine Martin (Nottingham University Hospitals NHS Trust, Diagnostic Laboratory Scientist); Katrina Prescott (Chapel Allerton Hospital, Recruiting Consultant Clinical Geneticist); Kay Metcalfe (St Mary’s Hospital & Central Manchester University Hospitals NHS Foundation Trust, Recruiting Consultant Clinical Geneticist); Kirsten McKay (Birmingham Women's Hospital, Diagnostic Laboratory Scientist); Kirsty Ambridge (Wellcome Trust Sanger Institute & University of Oxford, DDD Laboratory Team); Lara Cresswell (Leicester Royal Infirmary, Diagnostic Laboratory Scientist); Laura E. Mason (Wellcome Trust Sanger Institute & University of Oxford, DDD Laboratory Team); Laura Yates (Newcastle upon Tyne Hospitals NHS Foundation Trust, Patient recruitment and phenotyping); Leema Robert (Guy's Hospital, Recruiting Consultant Clinical Geneticist); Lily Islam (Birmingham Women’s Hospital, Patient recruitment and phenotyping); Linda Sneddon (Newcastle upon Tyne Hospitals NHS Foundation Trust & International Centre for Life, Research Nurse/Genetic Counsellor); Lisa Bradley (Belfast City Hospital, Patient recruitment and phenotyping); Liu He (Method development and data analysis) Lorraine Gaunt (St Mary’s Hospital & Central Manchester University Hospitals NHS Foundation Trust, Diagnostic Laboratory scientists); Louise Bourdon (Northwick Park Hospital & St Mark's Hospital, Diagnostic Laboratory scientists); Louise Brueton (Birmingham Women's Hospital, Recruiting Consultant Clinical Geneticist); Louise Nevitt (Sheffield Children's NHS Trust, Research Nurse/Genetic Counsellors); Louise Wilson (Great Ormond Street Hospital, Patient recruitment and phenotyping); Lucy Harrison (Princess Anne Hospital, Salisbury District Hospital, Research Nurse/Genetic Counsellor); Lucy Hilyard (Wellcome Trust Sanger Institute, Sample and data processing); Lucy Jenkins (Great Ormond Street Hospital, Diagnostic Laboratory Scientist); Lucy Raymond (Cambridge University Hospitals NHS Foundation Trust, Recruiting Consultant Clinical Geneticist); Lynn Greenhalgh (Royal Liverpool Children's Hospital, Recruiting Consultant Clinical Geneticist); Marc Tischkowitz (Cambridge University Hospitals NHS Foundation Trust, Patient recruitment and phenotyping); Margo Whiteford (Yorkhill Hospital, Recruiting Consultant Clinical Geneticist); Margriet van Kogelenberg (Wellcome Trust Sanger Institute & University of Oxford, DDD Analysis Team); Maria Bitner-Glindzicz (Great Ormond St Hospital, Patient recruitment and phenotyping); Mariella D’Alessandro (North of Scotland Regional Genetics Service, Research Nurse/Genetic Counsellor); Martin Pollard (Wellcome Trust Sanger Institute, Sample and data processing); Matthew E. Hurles (Wellcome Trust Sanger Institute & University of Oxford, DDD Management Team); Meena Balasubramanian (Sheffield Children's NHS Trust, Recruiting Consultant Clinical Geneticist); Melissa Lees (Great Ormond Street Hospital, Recruiting Consultant Clinical Geneticist); Melita Irving (Guy's Hospital, Recruiting Consultant Clinical Geneticist); Meriel McEntagart (University of London,

Recruiting Consultant Clinical Geneticist); Mervyn Humphreys (Belfast City Hospital, Diagnostic Laboratory Scientist); Michael Parker (Sheffield Children's NHS Trust, Recruiting Consultant Clinical Geneticist); Michael Parker (Wellcome Trust Sanger Institute & University of Oxford, DDD Ethics, Social Science and Policy Team); Michael Parker (Wellcome Trust Sanger Institute & University of Oxford, DDD Management Team); Michael Wright (Newcastle upon Tyne Hospitals NHS Foundation Trust & International Centre for Life, Recruiting Consultant Clinical Geneticist); Michael Yau (Guy's Hospital, Diagnostic Laboratory Scientist); Miranda Splitt (Newcastle upon Tyne Hospitals NHS Foundation Trust & International Centre for Life, Recruiting Consultant Clinical Geneticist); Miranda Squires (Chapel Allerton Hospital, Research Nurse/Genetic Counsellor); Mohnish Suri (Nottingham University Hospitals NHS Trust, Recruiting Consultant Clinical Geneticist); Mohsan Alvi (University of Oxford, Method development and data analysis); Moira Blyth (Chapel Allerton Hospital, Recruiting Consultant Clinical Geneticist); Morag N. Collinson (Princess Anne Hospital, Salisbury District Hospital, Diagnostic Laboratory scientists); Munaza Ahmed (Princess Anne Hospital, Salisbury District Hospital, Recruiting Consultant Clinical Geneticists); Muriel Holder (North West Thames Regional Genetics Centre, North West London Hospitals NHS Trust, The Kennedy Galton Centre and St Mark’s NHS Trust, Patient recruitment and phenotyping); Nadia Akawi (Wellcome Trust Sanger Institute & University of Oxford, DDD Analysis Team); Natalie Canham (Northwick Park Hospital & St Mark's Hospital, Recruiting Consultant Clinical Geneticist); Neeti Ghali (Northwick Park Hospital & St Mark's Hospital, Recruiting Consultant Clinical Geneticist); Netravathi Krishnappa (Wellcome Trust Sanger Institute & University of Oxford, DDD Laboratory Team); Nicola Cooper (Birmingham Women's Hospital, Recruiting Consultant Clinical Geneticist); Nicola Foulds, (Princess Anne Hospital, Salisbury District Hospital, Recruiting Consultant Clinical Geneticists); Nicola Ragge (Birmingham Women's Hospital, Recruiting Consultant Clinical Geneticist); Nicola Williams (Yorkhill Hospital, Diagnostic Laboratory Scientist); Nigel P. Carter (Wellcome Trust Sanger Institute & University of Oxford, DDD Management Team); Nora Shannon (Nottingham University Hospitals NHS Trust, Recruiting Consultant Clinical Geneticist); Norman Pratt (Ninewells Hospital, Diagnostic Laboratory Scientist); Oliver Quarrell (Sheffield Children’s NHS Trust, Patient recruitment and phenotyping); Paul Batstone (North of Scotland Regional Genetics Service, Diagnostic Laboratory Scientist); Paul Roberts (Chapel Allerton Hospital, Diagnostic Laboratory Scientist); Peter Ellis (Wellcome Trust Sanger Institute, WTSI Pipelines Staff); Peter Turnpenny (Royal Devon & Exeter Hospital, Recruiting Consultant Clinical Geneticists); Philip Greene (University of Edinburgh, Western General Hospital, Research Nurse/Genetic Counsellor); Philip Jones (Wellcome Trust Sanger Institute & University of Oxford, DDD Informatics Team); Pradeep Vasudevan (Leicester Royal Infirmary, Recruiting Consultant Clinical Geneticist); Rachel Harrison (Nottingham Regional Genetics Service, Patient recruitment and phenotyping); Raheleh Rahbari (Wellcome Trust Sanger Institute, Method development and data analysis); Ray Miller (Wellcome Trust Sanger Institute & University of Oxford, DDD Informatics Team); Richard Fisher (Newcastle upon Tyne Hospitals NHS Foundation Trust & International Centre for Life, Recruiting Consultant Clinical Geneticist); Richard Gibbons (Oxford Radcliffe Hospitals NHS Trust, Recruiting Consultant Clinical Geneticist); Richard Sandford (Cambridge University Hospitals NHS Foundation Trust, Recruiting Consultant Clinical Geneticist); Richard Scott (Great Ormond Street Hospital, Recruiting Consultant Clinical Geneticist); Rob Andrews, (Wellcome Trust Sanger Institute, WTSI Pipelines Staff); Rohan Taylor (University of London, Diagnostic Laboratory Scientist); Roldan Singzon, (Northwick Park Hospital & St Mark's Hospital, Research Nurse/Genetic Counsellor); Rose Hawkins (St Michael's Hospital, Research Nurse/Genetic Counsellor); Rosemarie Davidson (Yorkhill Hospital, Recruiting Consultant Clinical Geneticist); Rosemary Kelsell (Wellcome Trust Sanger Institute, Sample and data processing); Rosie O’Shea (Our Lady's Children's Hospital, Research Nurse/Genetic Counsellors); Ruby Banerjee (Wellcome Trust Sanger Institute & University of Oxford, WTSI FISH Team); Ruth Armstrong (Cambridge University Hospitals NHS Foundation Trust, Recruiting Consultant Clinical Geneticist); Ruth McGowan (North of Scotland Regional Genetics Service, Recruiting Consultant Clinical Geneticist); Ruth Newbury-Ecob (St Michael's Hospital,

Recruiting Consultant Clinical Geneticists); Saba Sharif, Mark Tein (Birmingham Women's Hospital, Recruiting Consultant Clinical Geneticist); Saeed Al-Turki (Wellcome Trust Sanger Institute & University of Oxford, DDD Analysis Team); Sahar Mansour (University of London, Recruiting Consultant Clinical Geneticist); Sally Ann Lynch (Our Lady's Children's Hospital, Recruiting Consultant Clinical Geneticist); Sally Davies (University Hospital Of Wales & Glan Clwyd Hospital, Recruiting Consultant Clinical Geneticist); Sandra Kazembe (Leicester Royal Infirmary, Research Nurse/Genetic Counsellor); Sandra Louzada Gomes Pereira (Wellcome Trust Sanger Institute & University of Oxford, WTSI FISH Team); Sara Widaa (Wellcome Trust Sanger Institute, WTSI Pipelines Staff); Sarah Edkins (Wellcome Trust Sanger Institute, WTSI Pipelines Staff); Sarah Everest (Royal Devon & Exeter Hospital, Research Nurse/Genetic Counsellor); Sarah Hewitt (Chapel Allerton Hospital, Diagnostic Laboratory Scientist); Sarah Smithson (St Michael's Hospital, Recruiting Consultant Clinical Geneticists); Sarah Wallwark (Cambridge University Hospitals NHS Foundation Trust, Patient recruitment and phenotyping); Sarah Wilcox (Cambridge University Hospitals NHS Foundation Trust, Research Nurse/Genetic Counsellor); Sarju Mehta (Cambridge University Hospitals NHS Foundation Trust, Recruiting Consultant Clinical Geneticist); Sebastian S. Gerety (Wellcome Trust Sanger Institute & University of Oxford, DDD Model Organisms); Shalaka Samant (North of Scotland Regional Genetics Service, Diagnostic Laboratory Scientist); Shane McKee (Belfast City Hospital, Recruiting Consultant Clinical Geneticist); Shehla Mohammed (Guy's Hospital, Recruiting Consultant Clinical Geneticist); Shelagh Joss (Yorkhill Hospital, Recruiting Consultant Clinical Geneticist); Sian Ellard (Royal Devon & Exeter Hospital, Diagnostic Laboratory Scientist); Sian Morgan (University Hospital Of Wales & Glan Clwyd Hospital, Diagnostic Laboratory Scientist); Siddhartha Banka (St Mary’s Hospital, Patient recruitment and phenotyping); Simon Brent (Wellcome Trust Sanger Institute, Sample and data processing); Simon Holden (Cambridge University Hospitals NHS Foundation Trust, Recruiting Consultant Clinical Geneticist); Sofia Douzgou (St Mary’s Hospital, Patient recruitment and phenotyping); Soo-Mi Park (Cambridge University Hospitals NHS Foundation Trust, Recruiting Consultant Clinical Geneticist); Stephen Clayton (Wellcome Trust Sanger Institute & University of Oxford, DDD Informatics Team); Stephen Hellens (Newcastle upon Tyne Hospitals NHS Foundation Trust & International Centre for Life, Diagnostic Laboratory Scientist); Stewart Payne (Northwick Park Hospital & St Mark's Hospital, Diagnostic Laboratory scientists); Stuart Aitken (University of Edinburgh, Method development and data analysis); Stuart Ingram (Sheffield Children's NHS Trust, Research Nurse/Genetic Counsellors); Sue Price (Oxford Radcliffe Hospitals NHS Trust, Recruiting Consultant Clinical Geneticist); Susan Clasper, (Oxford Radcliffe Hospitals NHS Trust, Diagnostic Laboratory Scientist); Susan Gribble (Wellcome Trust Sanger Institute & University of Oxford, DDD Laboratory Team); Susan Holder (Northwick Park Hospital & St Mark's Hospital, Recruiting Consultant Clinical Geneticist); Susan McNerlan (Belfast City Hospital, Diagnostic Laboratory Scientist); Susan Tomkins (Bristol Genetics Service, Patient recruitment and phenotyping); Susann Schweiger (Ninewells Hospital, Recruiting Consultant Clinical Geneticist); Suzannah J. Bumpstead (Wellcome Trust Sanger Institute, WTSI Pipelines Staff); Swati Naik (Birmingham Women’s Hospital, Patient recruitment and phenotyping); Tabib Dabir (Belfast City Hospital, Recruiting Consultant Clinical Geneticist); Tanya Bayzetinova (Wellcome Trust Sanger Institute & University of Oxford, DDD Laboratory Team); Tara Montgomery (Newcastle upon Tyne Hospitals NHS Foundation Trust & International Centre for Life, Recruiting Consultant Clinical Geneticist); Tarjinder Singh (Wellcome Trust Sanger Institute, Method development and data analysis); Tessa Homfray (University of London, Recruiting Consultant Clinical Geneticist); Tina Fendick (Guy's Hospital, Research Nurse/Genetic Counsellors); Tomas W. Fitzgerald (Wellcome Trust Sanger Institute & University of Oxford, DDD Analysis Team); Tomas W. Fitzgerald (Wellcome Trust Sanger Institute & University of Oxford, DDD Informatics Team); Trevor Cole (Birmingham Women's Hospital, Recruiting Consultant Clinical Geneticist); Una Maye (Royal Liverpool Children's Hospital, Diagnostic Laboratory Scientist); Uruj Anjum (University of London, Research Nurse/Genetic Counsellor); Usha Kini (Oxford Radcliffe Hospitals NHS Trust, Recruiting Consultant Clinical Geneticist); V. K. Ajith Kumar (Great Ormond St Hospital, Patient recruitment and

phenotyping); Victoria Harrison (Wessex Clinical Genetics Service, Patient recruitment and phenotyping); Victoria Murday (Yorkhill Hospital, Recruiting Consultant Clinical Geneticist); Vijaya Parthiban, (Wellcome Trust Sanger Institute & University of Oxford, DDD Analysis Team); Vinod Varghese (University Hospital of Wales & Glan Clywd Hospital, Patient recruitment and phenotyping); Virginia Clowes (North West Thames Regional Genetics Centre, North West London Hospitals NHS Trust, The Kennedy Galton Centre and St Mark’s NHS Trust, Patient recruitment and phenotyping); Vivienne McConnell (Belfast City Hospital, Recruiting Consultant Clinical Geneticist); Vivienne Sutton, (Royal Liverpool Children's Hospital, Diagnostic Laboratory Scientist); Volker Straub (Newcastle upon Tyne Hospitals NHS Foundation Trust, Institute of Human Genetics, International Centre for Life, Patient recruitment and phenotyping); Wayne Lam (University of Edinburgh, Western General Hospital, Recruiting Consultant Clinical Geneticists); Wendy D. Jones (Wellcome Trust Sanger Institute & University of Oxford, DDD Analysis Team); Yanick Crow (St Mary’s Hospital & Central Manchester University Hospitals NHS Foundation Trust, Recruiting Consultant Clinical Geneticist); Zara Skitt (St Mary’s Hospital & Central Manchester University Hospitals NHS Foundation Trust, Research Nurse/Genetic Counsellors); and Zosia Miedzybrodzka (North of Scotland Regional Genetics Service, Patient recruitment and phenotyping).
